# Supplementary material for: Social tolerance and role model diversity increase tool use learning opportunities across chimpanzee ontogeny
Source: Commun Biol. 2025 Mar 28;8:509. doi: 10.1038/s42003-025-07885-4 (PMC11953367; doi:10.1038/s42003-025-07885-4)
Supplement: Supplementary file 2 — Supplementary Information [file 42003_2025_7885_MOESM2_ESM.pdf]

Supplementary materials

Supplementary materials for the material and methods section

Table S1: List of the individuals studied with community, sex, mean age, observation time, and observation period.

ID	Community	Sex	Mean age (in years)	Observation time (in hours)	Observation period (in months)
20	South	Female	0.42	7.40	0
12	South	Female	0.53	10.08	1
13	East	Male	0.59	9.38	1
15	East	Male	0.67	1.73	0
5	South	Female	0.72	5.08	3
11	South	Male	0.92	53.32	11
10	South	Female	0.99	77.27	12
3	South	Male	1.87	74.19	13
27	South	Male	1.99	71.48	13
28	East	Female	2.13	90.79	14
51	North	Male	2.19	49.63	11
71	North	Female	2.42	46.08	6
19	East	Male	3.11	88.46	13
81	South	Female	3.33	67.92	14
87	South	Male	3.47	59.80	13
67	South	Male	3.52	57.01	13
22	East	Male	4.48	32.68	2
31	South	Male	4.51	71.74	13
73	East	Male	4.63	41.32	14
92	South	Female	4.65	90.45	14
61	East	Male	4.75	89.93	14
88	South	Male	4.78	65.01	12
38	East	Male	4.88	51.34	12
45	South	Female	5.25	52.87	10
6	South	Female	5.44	58.79	12
54	South	Male	5.57	61.38	12
59	North	Male	5.69	58.86	11
43	South	Female	6.49	45.35	12
55	North	Male	7.48	49.45	11
40	South	Male	7.60	38.15	6
72	South	Male	8.28	47.82	13
89	South	Female	8.69	55.08	11
46	South	Female	8.74	56.84	11
66	East	Female	9.06	58.34	13
57	North	Female	9.75	50.07	12

In model 1b and 2c, we used survival analyses to test: 1b) The effect of social interaction type on the latency to manipulate tools. 2c) The effect of extractive foraging attempt outcomes on the latency before the next peering event. In these two contexts we might not necessarily expect proportional hazards as 1) The initial action (social interaction or feeding attempt) conditions the outcome, and 2) the motivation to feed or learn, and the access to resources might decrease over time. We therefore compared how closely the uncensored data of the two models fitted a gamma, Weibull or a log normal distribution using the Bayesian information criterion (BIC), and visually compared it using a probability–probability plot (PP-plot) and a quantile-quantile plot (QQ-plot). To do this, we used the function “fitdist” from the r package “fitdistrplus”. For the two models, the BIC was much lower for the log-normal distribution. Additionally, visual inspection of QQ-plot and PP-plot suggested that our data better fitted the log normal distribution.

**Distribution**	**BIC Model 1b**	**BIC Model 2c**
Gamma	-3457.0	-4873.2
Weibull	-3509.8	-4778.3
Log-normal	**-3735.8**	**-5115.4**

Figure S1: Posterior Predictive Check of all the models used for the predictions.

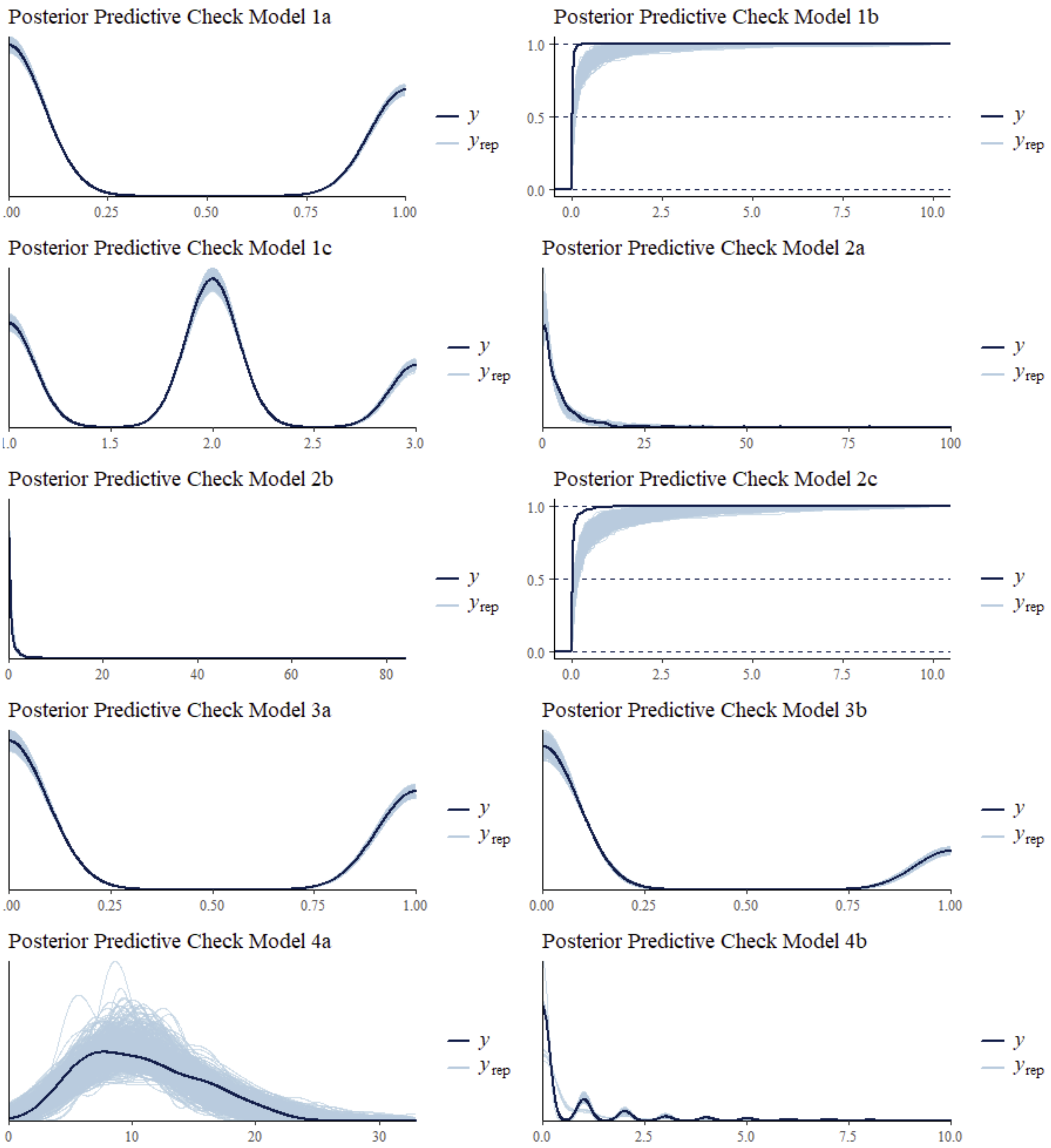

Table S2: List of the models and related predictions.

Model 1a: Effect of social interaction type on food transfer (Bernoulli model)

Prediction: Peering will elicit less food transfers than explicit solicitations (3001 events)

Model formula: Food transfer ~ Age + Age^2 + Sex + Maternal rank + Group + Maternal relatedness + Type of foraging*Type of solicitation + Monopolizability + (1+Age+Age^2+Soliway+Type of foraging+Monopolizability|Focal) + (1+Age+Age^2+Soliway+Type of foraging+Monopolizability|Mother) + (1|Date)

Model 1b: Effect of interaction type on tool manipulation (Log-normal model)

Prediction: Peering will be more quickly followed by tool manipulation than explicit solicitations (856 events)

Model formula: Time | cens (Censor) ~ Age + Direction + Sex + Party size + Group + Type of interaction + Food transfer + (1+ Party size +Type of interaction+Direction+Food transfer|Focal) + (1+Party size+Type of interaction+Direction+Food transfer|Mother) + (1|Date)

Model 1c: Effect of task complexity on type of interaction (Categorical model)

Prediction: Peering will be proportionally more employed for complex task than explicit solicitations (3102 events)

Model formula: Type of interaction ~ Age + Age^2 + Sex + Maternal relatedness + Maternal rank + Type of foraging + Group + (1+Type of foraging+Age+Age^2|Focal) + (1+Type of foraging+Age+Age^2|Mother) + (1|Date)

Model 2a: Effect of age on peering rate (Negative binomial model)

Prediction: Peering behaviour will peak during development and persist after weaning (570 events)

Model formula: Number of peering ~ Sex*Age + Sex*Age^2 + Party size + Group + Maternal rank + (1+Age +Age^2+Party size|Focal) + (1+Age+Age^2+Party size |Mother) + offset (log(Observation time))

Model 2b: Effect of task complexity on peering rate (Poisson model)

Prediction: Processing of extractive foraging and monopolizable resources will elicit more peering (2697 events)

Model formula: Time | cens (Censor) ~ Success + Food type + Sex + Age + Age^2 + Party size + Group + Maternal rank + (1+Party size|Focal) + (1+Party size|Mother) + (1|Date)

Model 2c: Effect of individual success on peering probability (Log-normal model)

Prediction: Failures in feeding attempts will reduce the latency before the next peering event. (1280 events)

Model formula: Type of interaction ~ Age + Age^2+ Sex + Kin + Maternal rank + Type of foraging + Group + (1+Type of foraging+Age+Age^2|Focal) + (1+Type of foraging+Age+Age^2|Mother) + (1|Date)

Model 3a: Effect of age on reliance on mother (Bernoulli model)

Prediction: Mothers will be more likely to be observed early in development (2024 events)

Model formula: Role model choice ~ Age*Party size + Sex + Group + Presence of sibling + Maternal rank + (1+Party size+Age |Focal) + (1+Party size+Age|Mother) + (1|Date)

Model 3b: Effect of task complexity on reliance on mother (Bernoulli model)

Prediction: Mothers will be more likely to be observed for complex tasks (1204 events)

Model formula: Role model choice ~ Age*Type of foraging + Party size*Type of foraging + Sex + Monopolizability + Group + Maternal rank + Presence of sibling + (1+Age+Type of foraging+Party size+Monopolizability|Focal) + (1+Age+Type of foraging+Party size+Monopolizability|Mother) + (1|Date)

Model 4a: Effect of age on the number of peered role models (Poisson model)

Prediction: Number of observed role models will peak during development (30 events)

Model formula: Number of observed role models ~ Age + Age^2 + Group + offset (log(Time))

Model 4b: Effect of role models age and tolerance on the number of peering (Negative binomial model)

Prediction: Older and more tolerant role models will be more often observed (1082 events)

Model formula: Number of peering per role model ~ Age peerer*log(Age receiver) + Age peerer*Maternal kin + Age peerer*Dyadic association + Age peerer^2 + Group + Sex receiver*Sex peerer + Time in close proximity + (1+Sex receiver+log(Age receiver)+Dyadic association+Time in close proximity|Focal) + (1+Age peerer+Age peerer^2+Sex peerer+Time in close proximity+Dyadic association|Receiver) + offset(log(Association time))

Supplementary materials for the result section

Table S3: Table showing the contrast between the levels not shown in the main text.

Model 1a: Effect of social interaction type on food transfer (Bernoulli model)

Term	Estimate	SE	95% CI	95% CI	89% CI	89% CI
Group North[a]	0,17	0.59	-1.04	1.32	-0.78	1.10
**Receiver (Other)[d]**	**-3.27**	**0.28**	**-3.84**	**-2.74**	**-3.73**	**-2.83**
Extractive with tool[b]	0.35	0.35	-0.35	1.04	-0.23	0.90
**Peering[c]**	**-2.53**	**0.50**	**-3.54**	**-1.56**	**-3.34**	**-1.74**
**Extractive with tool[b]:Peering[c]**	**1.33**	**0.35**	**0.66**	**2.00**	**0.78**	**1.87**
Extractive with tool[b]:Peering+Explicit begging[c]	0.17	0.59	-1.04	1.32	-0.78	1.10

Model 1b: Effect of interaction type on tool manipulation (Log-normal model)

Term	Estimate	SE	95% CI	95% CI	89% CI	89% CI
Group North[a]	0.75	0.79	-0.84	2.27	-0.54	1.98
Peering[c]	0.02	0.42	-0.81	0.86	-0.66	0.70

Model 1c: Effect of task complexity on type of interaction (Categorical model)

Term	Estimate	SE	95% CI	95% CI	89% CI	89% CI
**Receiver (Other)[d]**	**2.90**	**0.19**	**2.53**	**3.28**	**2.60**	**3.20**
**Extractive with tools[b]**	**1.07**	**0.37**	**0.35**	**1.79**	**0.49**	**1.65**
Group North[a]	-1.12	0.74	-2.59	0.34	-2.30	0.06
Receiver (Other)[d]	-0.33	0.27	-0.87	0.17	-0.76	0.08
Extractive with tools[b]	*0.85*	*0.44*	*-0.05*	*1.68*	*0.15*	*1.53*
Group North[a]	-0.04	0.66	-1.31	1.25	-1.08	1.01

Model 2a: Effect of age on peering rate (Negative binomial model)

Term	Estimate	SE	95% CI	95% CI	89% CI	89% CI
Group North[a]	*-0.80*	*0.47*	*-1.70*	*0.14*	*-1.53*	*-0.06*

Model 2b: Effect of task complexity on peering rate (Poisson model)

Term	Estimate	SE	95% CI	95% CI	89% CI	89% CI
**Extractive with tools[b]**	**1.17**	**0.36**	**0.42**	**1.87**	**0.58**	**1.72**
Group North[a]	-0.79	0.51	-1.79	0.20	-1.58	0.01
Extractive with tool[b]:Age	-0.32	0.30	-0.89	0.30	-0.79	0.17
Extractive with tool[b]: Age^2	-0.11	0.21	-0.53	0.29	-0.45	0.23

Model 2c: Effect of individual success on peering probability (Log-normal model)

Term	Estimate	SE	95% CI	95% CI	89% CI	89% CI
Group North[a]	0.15	0.90	-1.62	1.92	-1.30	1.61

Model 3a: Effect of age on reliance on mother (Bernoulli model)

Term	Estimate	SE	95% CI	95% CI	89% CI	89% CI
Group North[a]	-0.25	0.57	-1.37	0.85	-1.14	0.65

Model 3b: Effect of task complexity on reliance on mother (Bernoulli model)

Term	Estimate	SE	95% CI	95% CI	89% CI	89% CI
Extractive with tools[b]	*1.15*	*0.60*	*-0.07*	*2.31*	*0.16*	*2.08*
Group North[a]	-0.16	0.91	-1.90	1.60	-1.59	1.31
Extractive with tool[b]:Age	-0.26	0.46	-1.19	0.64	-1.01	0.47
**Extractive with tool[b]:Party size**	**0.76**	**0.33**	**0.13**	**1.40**	**0.24**	**1.27**

Model 4a: Effect of age on the number of peered role models (Poisson model)

Term	Estimate	SE	95% CI	95% CI	89% CI	89% CI
Group North[a]	-0.29	0.18	-0.67	0.06	-0.59	0.00

Model 4b: Effect of role models age and tolerance on the number of peering (Negative binomial model)

Term	Estimate	SE	95% CI	95% CI	89% CI	89% CI
Group North[a]	-0.39	0.42	-1.22	0.43	-1.06	0.28

Results of our statistical models, including the dependent variables, the effects with associated estimated error and credible interval at 95 and 89% (respectively in bold or italic if they did not cross 0). ([a]Group South, [b]Non extractive foraging, [c]Peering + Explicit begging, [d]Mother as reference categories).

Figure S2: Effect of offspring age, task complexity and role model choices on food transfers

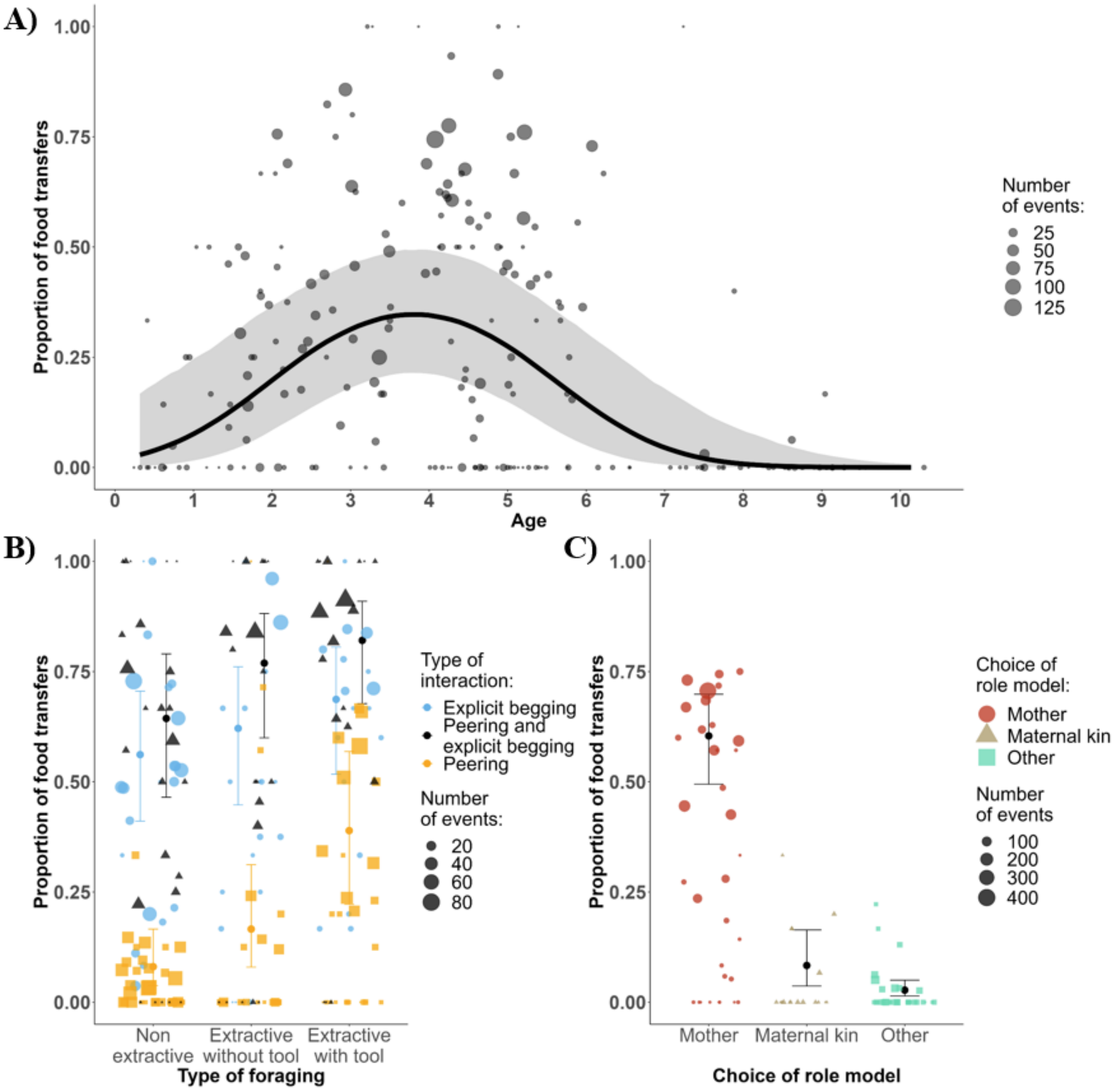

A) Effect of immatures age on the probability of food transfers following solicitations (model 1). B) Effect of the interaction between solicitation type and task complexity on the probability of food transfers. The regression lines from the Bayesian Regression model show a 95% credible interval. C) Effect of role model choices (mother, maternal kin or other) on the probability of food transfers following a solicitation (model 1). Each dot represents the proportion of food transfers per subject and age in month (A), per subject, type of social interaction and type of foraging (B), per subject and maternal relatedness (C).

Figure S3: Effect of receiver's identity and subject's age on the proportion of solicitations that include peering.

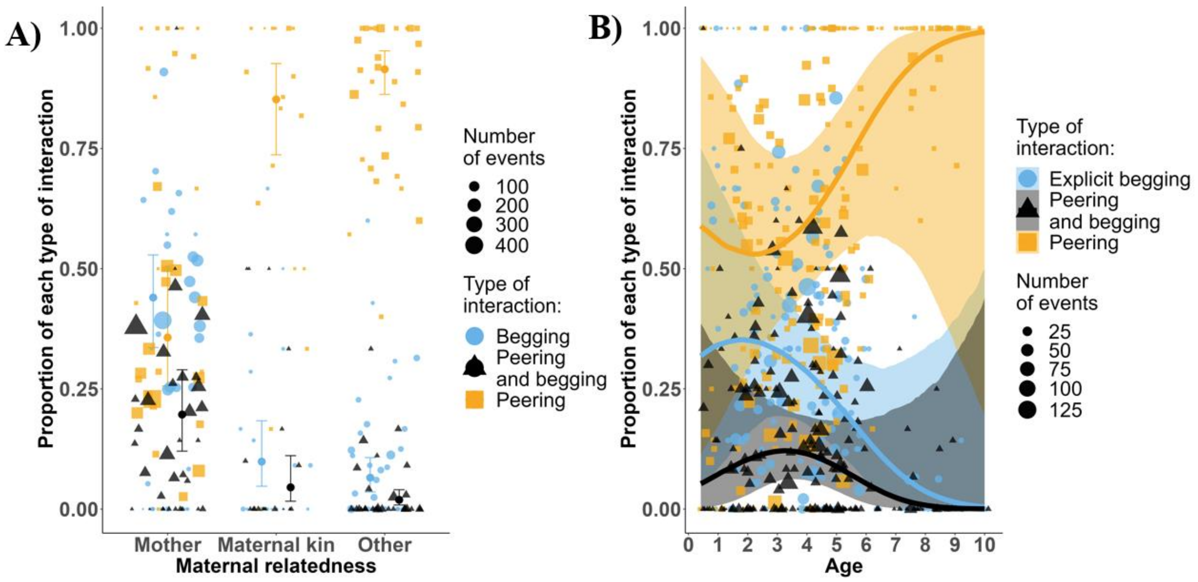

A) Effect of immatures age on the proportion of solicitations that are, or include, peering events (model 1c). B) Effect of role model choices (mother or not) on the proportion of solicitations that are, or include, peering events (model 1c). The regression lines from the Bayesian Regression model show a 95% credible interval. Each dot represents the proportion of each type of social interaction per subject and maternal relatedness of the role model (A), and per subject, type of social interaction and age (in months) (B).

Role model access during the development

During our focal follows, the average party size was 8.94 individuals of more than 4 years. Mothers were in the same party more than 98% of the time. At least one non-mother individual of more than 4 years was present 95% of the time.

Although party size was relatively consistent over the first decade of life, we expected social tolerance and access to the individuals could change during development.

We predicted that mothers would stay in close proximity to their offspring more than other role models during at least the first ten years of immatures development. To test the effect of age and maternal relationship on spatial proximity, we used a GLMM model with a Bernoulli distribution. For each proximity scan we tested the probability of a given individual present in the party composition to be in close proximity (<1m) (yes or no). As we expected that the probability for offspring to be in close proximity to the mother would decrease during development, we included an interaction between age and maternal relatedness (mother, maternal kin, other). We controlled for community, party-size, maternal rank and immatures' sex using fixed factors. We included random factors for the scan, the date, the identity of the focal and the identity of the partner to reduce pseudo-replication. Our model included 144 296 tests of proximity.

*Close proximity ~ Age*Maternal kin + Party size + Group + Sex + Maternal rank + (1|scan) + (1+Party size|DateFocal) + (1+Age+Party size|Focal) + (1+Age+Party size+Sex+Maternal rank|Partner) + (1+Party size|Dyad)*

We found that the probability of immatures to be in close proximity to a given individual decreased with age 95% CI [-0.54 -0.12] **(Table S4)** and this more for mothers than for other dyads 95% CI [-1.20; -0.63]. Additionally, immatures were overall more likely to be in close proximity to their mother 95% CI [1.98; 2.70] and to other maternal kin 95% CI [1.16; 1.69] than to other individuals.

These results support that mothers offer more spatial tolerance than other dyads, and this even relatively late in offspring development (towards 10 years). Interestingly, the average proportion of time spent in close proximity with all types of dyads tended to decrease during development, suggesting that immatures receive an overall higher level of spatial tolerance early in their development.

Table S4: Table of results of model testing the effect of immature age and maternal relatedness on close spatial proximity. Results of our statistical models, including the dependent variables, the effects with associated estimated error and credible interval 95 (in bold if they did not cross 0). ([a]Group East, [b]Maternal kin as reference categories).

**Model S1: Effect of age and maternal relatedness on time spent in close proximity** (Bernoulli model)				
**Term**	**Estimate**	**SE**	**95% CI**	**95% CI**
Intercept	-3.08	0.22	-3.52	-2.63
**Age**	**-0.33**	**0.11**	**-0.54**	**-0.12**
**Maternal relatedness (Mother)** [b]	**2.34**	**0.19**	**1.98**	**2.70**
**Maternal relatedness (Other)** [b]	**-1.42**	**0.14**	**-1.69**	**-1.16**
**Party size**	**-0.69**	**0.06**	**-0.76**	**-0.58**
**Group (North)** [a]	**-0.98**	**0.27**	**-1.51**	**-0.45**
Group (South) [a]	-0.17	0.20	-0.56	0.22
Sex (Male)	0.06	0.13	-0.20	0.33
Maternal rank	0.05	0.07	-0.10	0.20
**Age: Maternal relatedness (Mother)** [b]	**-0.91**	**0.14**	**-1.20**	**-0.63**
Age: Maternal relatedness (Other) [b]	0.08	0.10	-0.11	0.26

Figure S4: Proportion of time spent in close proximity per dyad depending on immature's age and maternal relatedness (model S1).

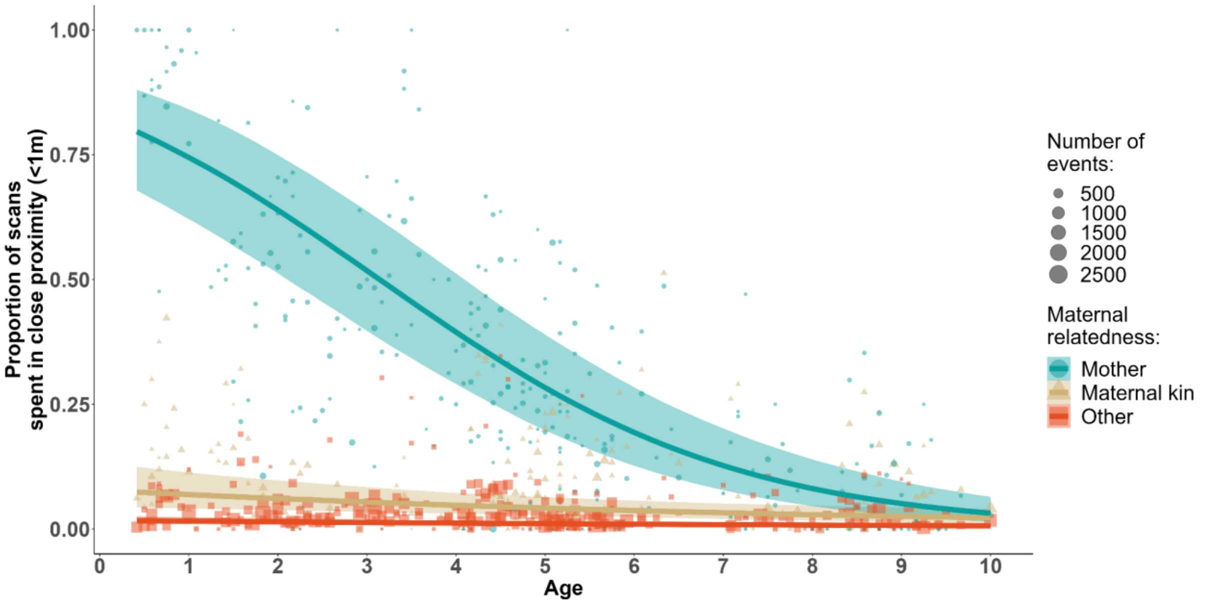

The regression lines from the Bayesian Regression model show a 95% credible interval. Each dot represents the number of tested scans per focal, age and maternal relatedness of the partner. Each dot represents the proportion of scans spend in close proximity (<1m) for each dyad.
