# Supplementary material for: Social tolerance and role model diversity increase tool use learning opportunities across chimpanzee ontogeny
Source: Commun Biol. 2025 Mar 28;8:509. doi: 10.1038/s42003-025-07885-4 (PMC11953367; doi:10.1038/s42003-025-07885-4)
Supplement: Supplementary file 3 — reporting-summary [file 42003_2025_7885_MOESM3_ESM.pdf]

nature portfolio

Corresponding author(s): Oscar Nodé-Langlois

Last updated by author(s): 2025/02/18

Reporting Summary

Nature Portfolio wishes to improve the reproducibility of the work that we publish. This form provides structure for consistency and transparency in reporting. For further information on Nature Portfolio policies, see our Editorial Policies and the Editorial Policy Checklist.

Statistics

For all statistical analyses, confirm that the following items are present in the figure legend, table legend, main text, or Methods section.

n/a	Confirmed	
☐	☒	The exact sample size (*n*) for each experimental group/condition, given as a discrete number and unit of measurement
☐	☒	A statement on whether measurements were taken from distinct samples or whether the same sample was measured repeatedly
☐	☒	The statistical test(s) used AND whether they are one- or two-sided<br>*Only common tests should be described solely by name; describe more complex techniques in the Methods section.*
☐	☒	A description of all covariates tested
☐	☒	A description of any assumptions or corrections, such as tests of normality and adjustment for multiple comparisons
☐	☒	A full description of the statistical parameters including central tendency (e.g. means) or other basic estimates (e.g. regression coefficient) AND variation (e.g. standard deviation) or associated estimates of uncertainty (e.g. confidence intervals)
☒	☐	For null hypothesis testing, the test statistic (e.g. *F*, *t*, *r*) with confidence intervals, effect sizes, degrees of freedom and *P* value noted<br>*Give P values as exact values whenever suitable.*
☐	☒	For Bayesian analysis, information on the choice of priors and Markov chain Monte Carlo settings
☐	☒	For hierarchical and complex designs, identification of the appropriate level for tests and full reporting of outcomes
☐	☒	Estimates of effect sizes (e.g. Cohen's *d*, Pearson's *r*), indicating how they were calculated

Our web collection on statistics for biologists contains articles on many of the points above.

Software and code

Policy information about availability of computer code

Data collection	We collected this data using the CyberTracker software (v.3.527).
Data analysis	Data preparation and statistical analyses were conducted in R 4.3.0 using the RStudio Interface (RStudio 228 Team 2020).

For manuscripts utilizing custom algorithms or software that are central to the research but not yet described in published literature, software must be made available to editors and reviewers. We strongly encourage code deposition in a community repository (e.g. GitHub). See the Nature Portfolio guidelines for submitting code & software for further information.

Data

Policy information about availability of data

All manuscripts must include a data availability statement. This statement should provide the following information, where applicable:

- Accession codes, unique identifiers, or web links for publicly available datasets
- A description of any restrictions on data availability
- For clinical datasets or third party data, please ensure that the statement adheres to our policy

The data and the codes used during the current study are available on zenodo at the following link: https://doi.org/10.5281/zenodo.13951539

Research involving human participants, their data, or biological material

Policy information about studies with human participants or human data. See also policy information about sex, gender (identity/presentation), and sexual orientation and race, ethnicity and racism.

Reporting on sex and gender	*Use the terms sex (biological attribute) and gender (shaped by social and cultural circumstances) carefully in order to avoid confusing both terms. Indicate if findings apply to only one sex or gender; describe whether sex and gender were considered in study design; whether sex and/or gender was determined based on self-reporting or assigned and methods used. Provide in the source data disaggregated sex and gender data, where this information has been collected, and if consent has been obtained for sharing of individual-level data; provide overall numbers in this Reporting Summary. Please state if this information has not been collected. Report sex- and gender-based analyses where performed, justify reasons for lack of sex- and gender-based analysis.*
Reporting on race, ethnicity, or other socially relevant groupings	*Please specify the socially constructed or socially relevant categorization variable(s) used in your manuscript and explain why they were used. Please note that such variables should not be used as proxies for other socially constructed/relevant variables (for example, race or ethnicity should not be used as a proxy for socioeconomic status). Provide clear definitions of the relevant terms used, how they were provided (by the participants/respondents, the researchers, or third parties), and the method(s) used to classify people into the different categories (e.g. self-report, census or administrative data, social media data, etc.) Please provide details about how you controlled for confounding variables in your analyses.*
Population characteristics	*Describe the covariate-relevant population characteristics of the human research participants (e.g. age, genotypic information, past and current diagnosis and treatment categories). If you filled out the behavioural & social sciences study design questions and have nothing to add here, write "See above."*
Recruitment	*Describe how participants were recruited. Outline any potential self-selection bias or other biases that may be present and how these are likely to impact results.*
Ethics oversight	*Identify the organization(s) that approved the study protocol.*

Note that full information on the approval of the study protocol must also be provided in the manuscript.

Field-specific reporting

Please select the one below that is the best fit for your research. If you are not sure, read the appropriate sections before making your selection.

☐ Life sciences ☐ Behavioural & social sciences ☒ Ecological, evolutionary & environmental sciences

For a reference copy of the document with all sections, see nature.com/documents/nr-reporting-summary-flat.pdf

Life sciences study design

All studies must disclose on these points even when the disclosure is negative.

Sample size	*Describe how sample size was determined, detailing any statistical methods used to predetermine sample size OR if no sample-size calculation was performed, describe how sample sizes were chosen and provide a rationale for why these sample sizes are sufficient.*
Data exclusions	*Describe any data exclusions. If no data were excluded from the analyses, state so OR if data were excluded, describe the exclusions and the rationale behind them, indicating whether exclusion criteria were pre-established.*
Replication	*Describe the measures taken to verify the reproducibility of the experimental findings. If all attempts at replication were successful, confirm this OR if there are any findings that were not replicated or cannot be reproduced, note this and describe why.*
Randomization	*Describe how samples/organisms/participants were allocated into experimental groups. If allocation was not random, describe how covariates were controlled OR if this is not relevant to your study, explain why.*
Blinding	*Describe whether the investigators were blinded to group allocation during data collection and/or analysis. If blinding was not possible, describe why OR explain why blinding was not relevant to your study.*

Behavioural & social sciences study design

All studies must disclose on these points even when the disclosure is negative.

Study description	*Briefly describe the study type including whether data are quantitative, qualitative, or mixed-methods (e.g. qualitative cross-sectional, quantitative experimental, mixed-methods case study).*
Research sample	*State the research sample (e.g. Harvard university undergraduates, villagers in rural India) and provide relevant demographic information (e.g. age, sex) and indicate whether the sample is representative. Provide a rationale for the study sample chosen. For studies involving existing datasets, please describe the dataset and source.*

Sampling strategy	*Describe the sampling procedure (e.g. random, snowball, stratified, convenience). Describe the statistical methods that were used to predetermine sample size OR if no sample-size calculation was performed, describe how sample sizes were chosen and provide a rationale for why these sample sizes are sufficient. For qualitative data, please indicate whether data saturation was considered, and what criteria were used to decide that no further sampling was needed.*
Data collection	*Provide details about the data collection procedure, including the instruments or devices used to record the data (e.g. pen and paper, computer, eye tracker, video or audio equipment) whether anyone was present besides the participant(s) and the researcher, and whether the researcher was blind to experimental condition and/or the study hypothesis during data collection.*
Timing	*Indicate the start and stop dates of data collection. If there is a gap between collection periods, state the dates for each sample cohort.*
Data exclusions	*If no data were excluded from the analyses, state so OR if data were excluded, provide the exact number of exclusions and the rationale behind them, indicating whether exclusion criteria were pre-established.*
Non-participation	*State how many participants dropped out/declined participation and the reason(s) given OR provide response rate OR state that no participants dropped out/declined participation.*
Randomization	*If participants were not allocated into experimental groups, state so OR describe how participants were allocated to groups, and if allocation was not random, describe how covariates were controlled.*

Ecological, evolutionary & environmental sciences study design

All studies must disclose on these points even when the disclosure is negative.

Study description	In this study, we studied the development of observational social learning opportunities in wild chimpanzees. We used peering behaviour (close observation of a conspecific) as a proxy of social learning opportunities. Our data were observational (no experiments), and were analysed using Bayesian generalised linear mixed models.
Research sample	We studied three communities of wild chimpanzees Pan troglodytes verus, between January 2021 and April 2023. As our study focused on immatures, we collected data on 35 individuals between 0 and 11 years (20 males, 15 females). All non-orphan individuals of this age range were followed. We used half-day focal sampling, and recorded a total of 1873 observations hours during 570 focal half-days. We chose the order of the focal pseudo-randomly, with priority given to less observed individuals to balance observation hours across subjects.
Sampling strategy	We used half-day focal sampling, and recorded a total of 1873 observations hours during 570 focal half-days. We chose the order of the focal pseudo-randomly, with priority given to less observed individuals to balance observation hours across subjects.
Data collection	The data were collected by 4 observers who conducted inter-reliability tests. We conducted half way focal and chose the order of the focal pseudo-randomly, with priority given to less observed individuals to balance observation hours across subjects.
Timing and spatial scale	Data were collected continuously between between January 2021 and April 2023 in the Tai Nationnal Park (5°45 N, 7°7 W).
Data exclusions	As data collected were used for another study and that mothers were used as a main role models, we did not include orphans in the data collection. No data were excluded in the analysis (except for one that required bigger sample size model 4a). Procedure is detailed in the method.
Reproducibility	The study involved no experiments, but were collected in three different communities. The data and the code for the analysis conducted are provided.
Randomization	The study involved no experiments. The observers did inter-observers reliability tests and could switch between chimpanzees' groups.
Blinding	The data were collected by several observers that had to pass inter-reliability tests. However, there was no blinding as observers had to be able to successfully identify animals in order to collect the data.

Did the study involve field work? ☒ Yes ☐ No

Field work, collection and transport

Field conditions	The data were collected in the Taï National Park (Ivory Coast). Altitudes vary from 80 meters (260 ft) to 396 meters (1,299 ft). The mean annual rainfall was of 2,200 millimetres, falling from March/April to July, with a shorter wet season in September to October. The temperature fluctuates between 25 to 35 °C (77 to 95 °F). The relative humidity is of 85%.
Location	The study site is located in Côte d'Ivoire (5°45 N, 7°7 W). Elevation across the study site varies between 115 and 287 m.
Access & import/export	The study was permitted by the Ministère de l'Enseignement Supérieur et de la Recherche Scientifique and the Ministère de Eaux et Fôrets in Côte d'Ivoire, and the Office Ivoirien des Parcs et Réserves (Wittig/006/MESRS/DGRI).

Disturbance	This study was observational. To prevent risks of disease transmissions, researchers observed a 5 days quarantine before entering at the field site and respected strict hygienic rules. Researcher observed the chimpanzees at a minimum distance of 7m and wear masks while being in the forest.

Reporting for specific materials, systems and methods

We require information from authors about some types of materials, experimental systems and methods used in many studies. Here, indicate whether each material, system or method listed is relevant to your study. If you are not sure if a list item applies to your research, read the appropriate section before selecting a response.

Materials & experimental systems

n/a	Involved in the study
☒	☐ Antibodies
☒	☐ Eukaryotic cell lines
☒	☐ Palaeontology and archaeology
☐	☒ Animals and other organisms
☒	☐ Clinical data
☒	☐ Dual use research of concern
☒	☐ Plants

Methods

n/a	Involved in the study
☒	☐ ChIP-seq
☒	☐ Flow cytometry
☒	☐ MRI-based neuroimaging

Animals and other research organisms

Policy information about studies involving animals; ARRIVE guidelines recommended for reporting animal research, and Sex and Gender in Research

Laboratory animals	The study did not involve laboratory animals.
Wild animals	This study was observational and involved three communities of wild chimpanzees Pan troglodytes verus. 35 immatures (<11 years) were followed. To prevent risks of disease transmissions, researchers observed a 5 days quarantine before entering at the field site and respected strict hygienic rules. Researcher observed the chimpanzees at a minimum distance of 7m and wear masks while being in the forest.
Reporting on sex	Our finding applies to both sexes. Our study involved 15 females and 20 males, for which the sex was visually inferred. The sex of the studied individuals was reported in the data and was controlled for in the analysis when relevant. Only one analysis did not include sex because there was a small sample size and no strong effect were observed in other models.
Field-collected samples	The study did not involved samples collected in the field.
Ethics oversight	Research at the Taï Chimpanzee Project is using non-invasive methods and were approved by the “Ethikrat” of the Max Planck Society (04/08/2014).

Note that full information on the approval of the study protocol must also be provided in the manuscript.

Plants

Seed stocks	*Report on the source of all seed stocks or other plant material used. If applicable, state the seed stock centre and catalogue number. If plant specimens were collected from the field, describe the collection location, date and sampling procedures.*
Novel plant genotypes	*Describe the methods by which all novel plant genotypes were produced. This includes those generated by transgenic approaches, gene editing, chemical/radiation-based mutagenesis and hybridization. For transgenic lines, describe the transformation method, the number of independent lines analyzed and the generation upon which experiments were performed. For gene-edited lines, describe the editor used, the endogenous sequence targeted for editing, the targeting guide RNA sequence (if applicable) and how the editor was applied.*
Authentication	*Describe any authentication procedures for each seed stock used or novel genotype generated. Describe any experiments used to assess the effect of a mutation and, where applicable, how potential secondary effects (e.g. second site T-DNA insertions, mosiacism, off-target gene editing) were examined.*
